# Ewing's sarcoma of the hip: A case report with no evidence of tumor recurrence and literature review

**DOI:** 10.1016/j.bonr.2021.101131

**Published:** 2021-09-22

**Authors:** Payam Mohammadhoseini, Samira Razzaghi, Mahdi Barazesh, Sajad Jalili

**Affiliations:** a Orthopaedics Department, School of Medicine, Ahvaz Jundishapour University of Medical Sciences, Ahvaz, Iran; b Department of Radiotherapy, School of Medicine, Ahvaz Jundishapur University of Medical sciences, Ahvaz, Iran; c School of Paramedical, Gerash University of Medical Science, Gerash, Iran

**Keywords:** Ewing's sarcoma, Event-free survival, Metastasis, Pelvis, Radiation therapy

## Abstract

**Background:**

Ewing's sarcoma (ES) of the hip and trochanteric region is a rare malignancy. The tumor has a poor prognosis due to the problems in early diagnosis and medical intervention.

**Case presentation:**

This paper reports a rare case of hip ES presented in a 34y/o female. The clinical, radiological, and histopathological features were all in favor of ES. Following treatment by neoadjuvant/adjuvant chemotherapy, and irradiation the patient is now with complete resolution of the tumor.

**Conclusion:**

The patient remained free of disease through 4 years of follow-up until now after diagnosis.

## 1 Introduction

Ewing's sarcoma (ES) is an infrequent skeletal tumor with a highly aggressive and heterogenic entity. The tumor is the second most common primary bone malignancies after osteosarcoma and chondrosarcoma (Sun et al., 2017). The tumor heterogeneity leads to its poor prognosis due to expansion of pre-existing sub-clonal populations consisting of resistant/sensitive cells under therapeutic selective pressure. As a result of this heterogeneity, the tumor bulk might include a diverse collection of cells harboring distinct molecular signatures and non-uniform distribution of genetically distinct tumor-cell subpopulations with differential levels of sensitivity to treatment (Dagogo-Jack and Shaw, 2018). This malignancy at first was described by James Ewing in 1921 as an osteolytic skeletal tumor consisting of round, undifferentiated form, and small cancer cells with rapid growth and highly metastatic ability (Anderson et al., 2020). The tumor originates from embryonic osteochondrogenic progenitor cells or a mesenchymal stem cell. One important simple reason for this is the concept that it predominantly involves children and young persons with peak incidences between the ages of 10 to 20 years. The diaphysis of long bones (femur, tibia, and humorous), ribs, and flat bones, such as scapula and pelvis, are the most commonly affected sites. The prevalence accounts for approximately 9–11% of primary bone sarcomas (Sun et al., 2017; Murphey et al., 2013) and approximately 90% of cases present themselves before the age of 20 (Lawlor and Sorensen, 2015). Involvement of proximal metaphysis of the tibia is rare with an incidence frequency of about 4–11% (Abdi and Hassanzadeh Taheri, 2015; Puri et al., 2012).

The average delay from onset of symptoms to the diagnosis is about eight months (Strange et al., 2013). Immunohistochemistry (IHC) and molecular assays for specific tumor markers seem to be the main method of diagnosis. IHC proposes reliable diagnostic support such as specific staining for vimentin and CD99. The high expression of the selected markers is highly efficient in Ewing sarcoma diagnosing from osteosarcoma. Leukemia and lymphoma cell markers such as CD45, and muscle desmin are negative for this malignancy. Clinically, Ewing's sarcoma may present itself as a localized, painful mass or systemic symptoms such as fever, malaise, weight loss, leukocytosis, and increased erythrocyte sedimentation rate (ESR), and maybe mistaken with osteomyelitis (Henninger et al., 2013). ES has the most unfavorable prognosis among all primary musculoskeletal tumors. When the lesion is associated with systemic features of fever and weight loss as an indicator of metastasis and delay in diagnosis and effective therapy, the prognosis becomes even worse than average. Even with early intervention, patients with metastasis (usually lung and bone marrow) have approximately 20% chance of 5-year survival (Totadri et al., 2020). The therapeutic modalities for Ewing's sarcoma include local treatment (surgery and radiation therapy) and systemic therapy (adjuvant/ neoadjuvant chemotherapy). In metastatic or recurrent Ewing Sarcoma a combination of multidrug chemotherapy, wide tumor resection, and/or radiotherapy can improve local tumor control and prolong survival (Zöllner et al., 2021). The chemotherapeutic regimens used commonly include VAC (vincristine, adriamycin, and cyclophosphamide), followed by IE (ifosfamide and etoposide) (Huang and Lucas, 2011).

We herein introduced a 34y/o female case of ES arisen in her left hip with no evidence of recurrence following treatment. Furthermore, a review of pertinent literature was done to make the clinicians aware of the clinical and histopathological spectrum of this rare tumor.

## 2 Case presentation

**Fig. 1:**
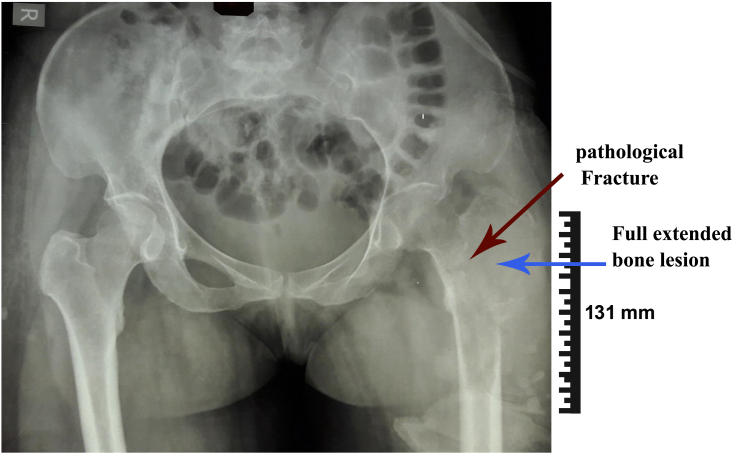
Plain radiograph of the pelvic demonstrated a pathological fracture of femur, large bone lesion and involvement of surrounded soft tissues in the presented patient.

The case was a 34y/o female developed with left hip pain and inability to weight-bearing after simple falling while walking. Thus her weight-bearing was impaired severely. Following informed consent, she was visited at the Orthopedics department and complained of obscure hip pain for six past months. She had been visited previously by physicians several times and treated as muscle spasm and low back pain. Her history was unremarkable, and the patient did not mention any positive past medical, surgical, or drug prescription history. In addition, there were no signs of fever and weight loss. Physical examination detected hip tenderness, and restricted range of motion (ROM). The neurovascular examination was normal. However, in obtained plain radiographs a pathologic fracture of the proximal femur, destructive bone mass, and soft tissue involvement were determined (Fig. 1).

**Fig. 2:**
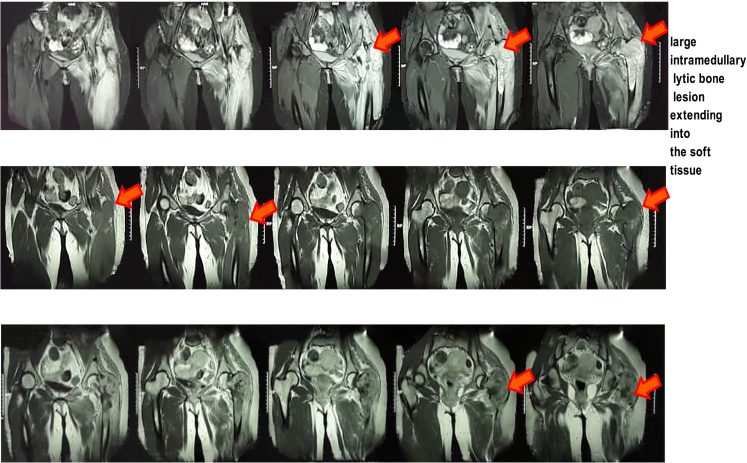
MRI of the pelvis showed a complex irregular border T1/T2 signal intensity, intramedullary lesion. The mass had a 131 × 70 mm × 60 mm size compressing the left piriformis muscle posteriorly as indicated by orange arrows. The tumor had heterogeneous enhancement at diaphysis and metaphysis of proximal part of left femur with disruption of cortex and infiltration to adjacent anterior and posterior muscle compartment. There was no involvement of neurovascular bundle.

The clinical and radiological assessment suggested a destructive bone lesion with a suspicion of malignancy. Then the patient was admitted to our institute at Jundishapour University of Medical Sciences, Ahwaz, Iran for further evaluation. Tumor follow-up was started and necessary evaluations such as pelvic MRI, related lab tests, chest, and abdominal pelvic CT scan, bone mass densitometry, and whole body scan were done. The results of lab tests including Hb, PLT, WBC, Ca, P, BS, TFT, LFT, BUN, CRE, ESR, PTH were in the normal range and viral markers were also negative. Besides, the obtained results of several measured specific bone turnover markers including serum bone-specific alkaline phosphatase (BSAP) (79.69 ± 3.9 U/L), and osteocalcin (23.58 ± 5.1 ng/mL) demonstrated a high level in serum of the patient. The obtained MRI images of the pelvis showed a large mass with a 131 ∗ 70 mm size and irregular border at T1/T2 intermediate signal intensity. Furthermore, MRI revealed an intramedullary lesion with heterogeneous enhancement at diaphysis and metaphysis of the proximal part of left femur and infiltration to adjacent anterior and posterior muscle compartment. There was no involvement of neurovascular bundle enhancement at diaphysis and metaphysis of the left femur proximal part (Fig. 2).

**Fig. 3:**
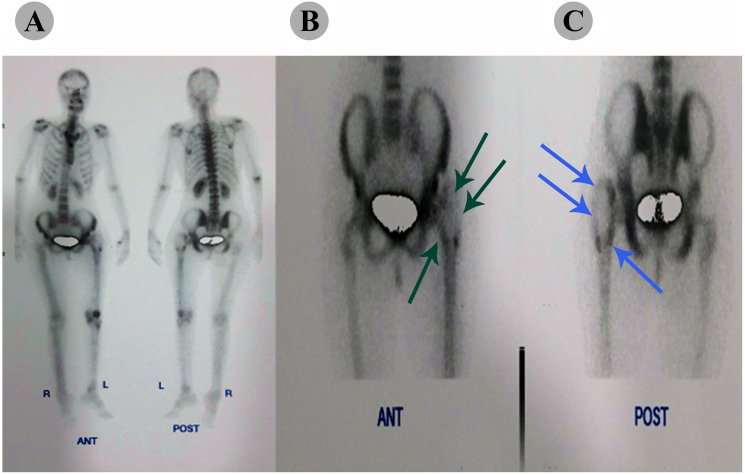
(A) A technetium bone scan showed increased uptake of radiotracer at the periphery of the lesion. (B) An anterior view of the left femur showed cortical erosion and Codman's triangle as indicated by green arrows. (C) A posterior view provided evidence of the tumor in a subperiosteal location and an intact medullary cavity as indicated by blue arrows. No osseous or cartilaginous matrix was found, and there was involvement of the surrounding soft tissue.

**Fig. 4:**
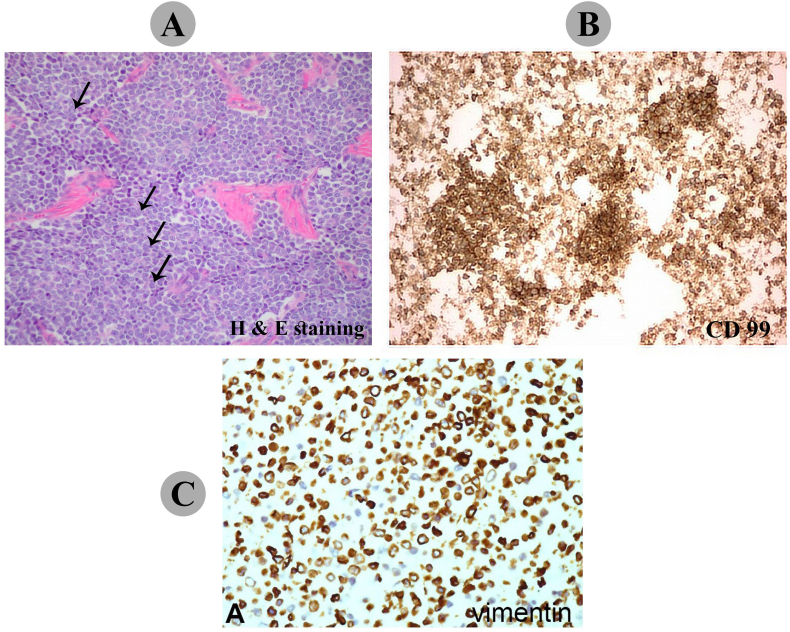
A) Histological section showed sheets of small round cells with large nuclei, peripheral ring of cytoplasm, and scanty stroma as indicated by arrow (H & E stain, 40 × 10 magnification). B and C) Immunohistochemistry (IHC) analysis showed CD99 (B) and vimentin (C) high expression in cell surface and cytoplasm of tumor cells respectively (40 × 10 magnification). Tissue biopsy revealed a small, round blue cell neoplasm. IHC stains were positive for both vimentin and CD99.

MRI scans (Siemens, MAGNETOM Espree model, 1.5 T, Erlangen, Germany) showed an expansion of the subperiosteal space, without spread to the underlying cortex. Whole body scan with Technetium-99m (TC_99m), demonstrated a hypoactive expanded bony lesion in left trochanteric region without involvement of other sites (Fig. 3). Therefore, the patient was diagnosed without evidence of metastatic disease as determined by staging chest radiographs, chest computed tomography scans, and bone scans. Following initial evaluation, the patient underwent open surgical biopsy from both the bone and soft tissue and was sent for pathological evaluation. Microscopic examination revealed an undifferentiated tumor consisting of small, round blue tumor cells with hyperchromatic round to oval shaped nuclei in favor of Ewing's sarcoma and recommended for immune histochemical (IHC) study for CD99, CD45, vimentin, and desmin markers analysis. The IHC findings showed the tumor cells were strongly positive for CD99 and vimentin and negative for CD45 and desmine, which also implies Ewing's sarcoma (Fig. 4).

The patient was referred to an oncologist and so decided for adjuvant chemotherapy regimen, and radiotherapy without surgical resection, and closed follow-up. She was then treated with 7 cycles of adjuvant chemotherapy regimen consisted of VAC (vincristine 2 mg/m^2^, Adriamycin 75 mg/m^2^, and cyclophosphamide 1200 mg/m^2^) alternating with IE (ifosfamide 1800 mg/m^2^ and etoposide 100 mg/m^2^) for 6 months. Then, radiation therapy consisted of 5400 cGy (180 cGy/fraction) was added to chemotherapy for local control of the tumor metastasis. The patient until now (4 years later) has remained alive without evidence of recurrence or metastasis.

**Fig. 5:**
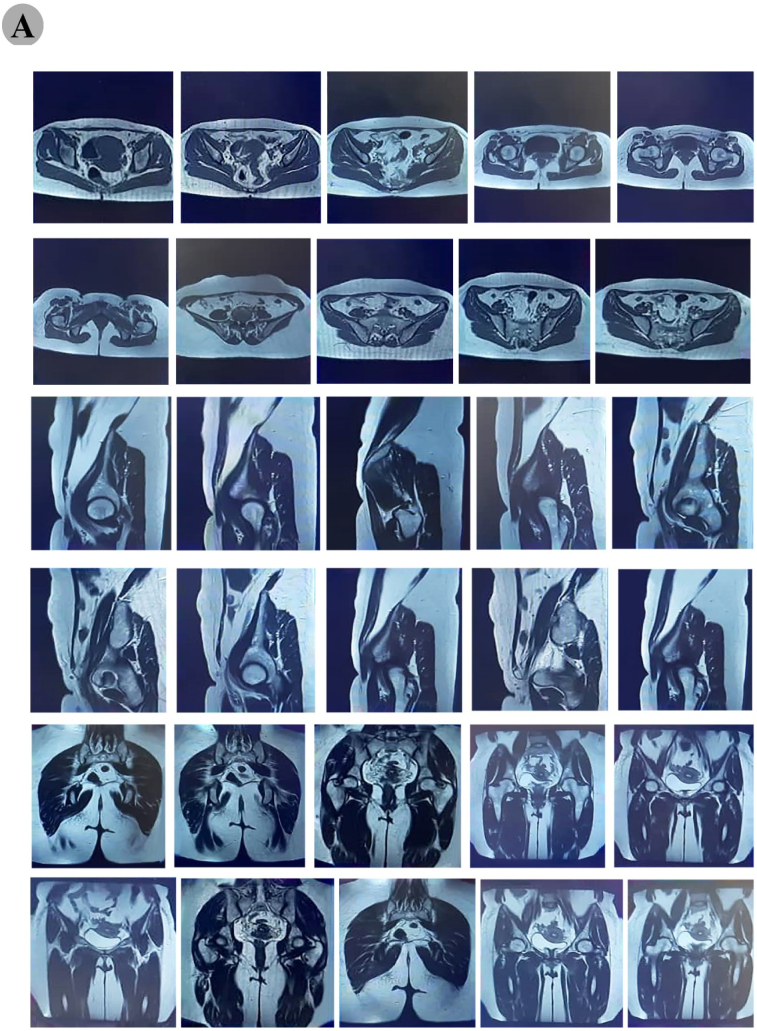

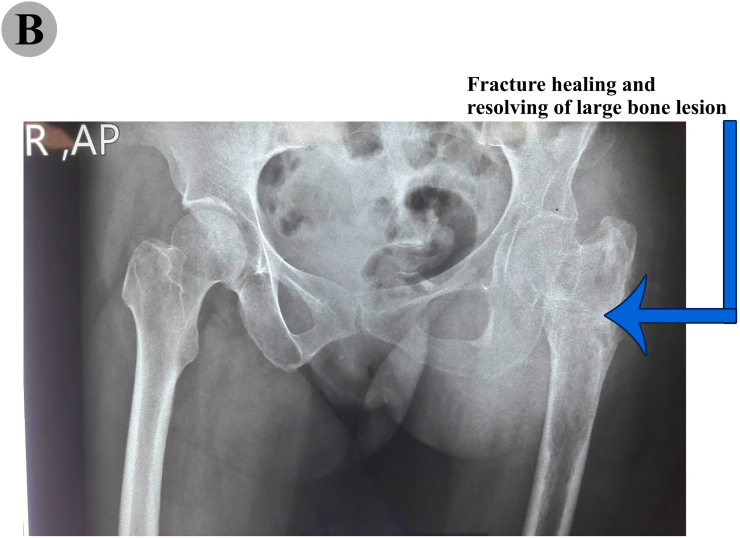
(A) T1/T2 weighted image of pelvis and left proximal part of the femur. The MRI scans showed a normal sub-periosteal space, without evidence of mass and no involvement of surrounded soft tissue and underlying cortex following treatment. 5. (B) An anteroposterior (AP) radiograph of the pelvic post combined treatment with chemotherapy and irradiation demonstrated fracture healing and no evidence of mass and involvement of surrounded soft tissues.

**Fig. 6:**
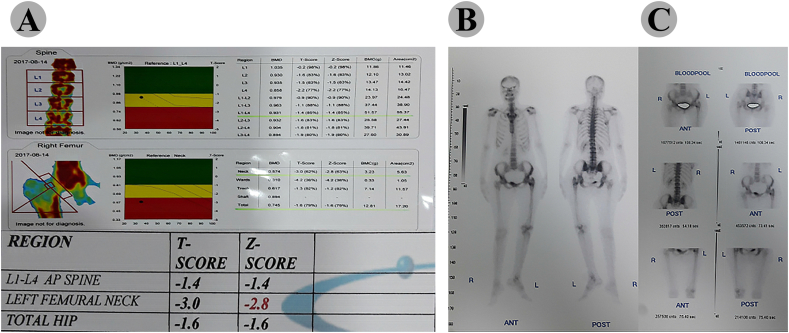
A) The result of bone mass densitometry (DEXA scan) indicated clear decrease in bone mass of proximal femur without reduced BMD in other sites including spine and total hip. B and C) Technetium bone scan following tri-modality therapy. No additional areas of abnormal radiotracer uptake were seen. A sagittal view provided evidence of the no tumor existence in a sub-periosteal location and an intact medullary cavity. No osseous or cartilaginous matrix was found, and there was no involvement of the surrounding soft tissue.

During the chemo-radiotherapy period, the patient was under closed follow up with lab and radiographic imaging. The follow-up images (bone scan, MRI, plain radiograph and CT scan) were done based on the recommendations of the CESS/LESS groups that proposed a defined timing of different imaging procedures (Heinemann et al., 2017). Follow-up imaging in serial radiographs after completion of the therapy showed good improvement and no evidence of metastasis was visible. Hip X-ray was done and demonstrated that the tumor was disappeared quietly and fracture was healed (Fig. 5B). Eventually, the chemo-radio treatment lasted for 12 months and the patient health status was excellent and remained asymptomatic and disease-free. Furthermore, Chest and abdominopelvic CT scan were negative for probable metastasis and involvement of any other sites. The last new MRI of the hip (3.8 years after diagnosis) was done that demonstrated no evidence of retained soft tissue involvement or bone tumor (Fig. 5A). The patient was referred for follow-up bone scan. The last one was taken 3.8 years later and result was negative for any bone involvement (Fig. 6B and C). The quantified bone turnover markers i.e. BSAP, and osteocalcin identified a normal range after completion of treatment. Following the chemotherapy and radiotherapy, bone mass densitometry (BMD) showed a clear decrease in bone mass of proximal femur (Fig. 6A). However, as the result of dual energy x-ray absorptiometry (DEXA) scan indicated (Fig. 6A), other sites including spine and total hip did not exhibit reduced BMD. The patient was satisfied, with no pain in rest or walking, some degree of limping due to mal-union appeared that patient was suggested for corrective osteotomy that refused.

## 3 Discussion

Ewing sarcoma (ES) is one tumor type of what is now regarded to be a category called Ewing sarcoma family of tumors (ESFT), which comprises peripheral primitive neuroectodermal tumors (PNETs) and Askin tumors. Many researchers have opinioned it as a different nature and the argument continued (Gongidi et al., 2014). Initial ES, usually engages early childhood or adolescence at first two decades of life and scarcely happens in adulthood and demonstrates slightly the male preponderance with a male to female incidence ratio of 1.5:1 (Raymond, 2020). This tumor has an infrequent incidence that involves bones and soft tissue surrounding the bone. The tumor fatality rate is extremely high (65–80%) when cured with surgery or radiation therapy alone for local control of the disorder (Fan et al., 2017). During the previous few decades improvement in chemotherapy, surgery, and beam irradiation therapy have ameliorated the prognosis of patients with ES (Kyriazoglou et al., 2019). Tumors of the pelvis have a poorer prognosis when compared with other sites. Whether this is related to the challenge of achieving local control or the proximity to critical deep structures should be elucidated. On the other hand, wide pelvis surgeries are requesting for the surgeon and the patient because of the navigational difficulty in the pelvis, numerous muscle connections, and the proximity of the main blood vessels, nerves, and visceral organs (Mankin et al., 2004). In general, the therapeutic approach for ES includes the tri-modality chemotherapy regimens and radical resection, followed by local radiotherapy control. Aside from the patient characteristics, local control also affects outcomes (Choi et al., 2015).

In many non resectable Ewing sarcomas, radiation therapy is often used after chemotherapy to further kill cancer cells and control their growth. The surgical approach should be combined with chemotherapy and additional radiotherapy in cases involving a marginal resection and/or poor histological response. The limited number of patients above the age of 30 and the removal of these patients from the majority of trials render these findings difficult to generalize (Thévenin-Lemoine et al., 2018). In the current case presentation, diagnosed extra-osseous Ewing sarcoma was reported in a 34y/o female. Then, the patient was referred to our department subjected to open biopsy, and treatments including neoadjuvant schemes could be administered to her during follow up. At this age, the patient had no local recurrence in her left hip. The patient also received radiation therapy for local recurrence/metastasis control (total dose: 54 Gy).

The literature review revealed a few cases of pelvic Ewing sarcoma which were treated with several chemotherapeutic regimens. The heterogeneity of the used regimens reflects the rarity of the disease and the evolution of multi-agent chemotherapy for Ewing Sarcoma during the last few decades (Bhansali and Desai, 1963; Nesbit et al., 1990). ES Diagnosing can be challenging. From microscopic aspects, these tumors are constituted of uniform tiny round to oval cells with round nuclei containing fine chromatin and scant clear or eosinophilic cytoplasm. Utilizing data from clinical, radiological, pathological, and cytogenetic origins can provide valuable diagnostic information. CD99 is the most commonly reported marker associated with ES, reported in 31 of the 39 cases found in the literature. Other associated markers, but less specific, include neuron specific antigen, vimentin, and synaptophysin (Widhe and Widhe, 2000). In our case, IHC stains of her tumor cells were demonstrated a strong intensity for CD99 expression but negative for CD45 marker, and the tumor cells had similar morphology to those previously detected in Ewing sarcoma.

Prompt diagnosis, by multimodality approach, which includes appropriate imaging (bone scan with radioisotopes), biopsy (diagnostic or excision) is required to tailor appropriate treatment regimen for optimal profit. Collectively, these diagnostic techniques were critical in supporting the proper diagnosis of ES metastatic to the other sites (Nesbit et al., 1990).

In ES, bone serum markers are helpful to assess the tumor diffusion, the response to therapy, and survival. The osteoblastic lesions are manifested by high levels of serum bone turnover markers. High levels of these markers forecast poor prognosis. The utilization of bone markers in malignant tumors permits an early diagnosis, fast onset of therapy, restricted complications, and predict the risk of relapse. However, the sensitivity of these markers is very low, proposing that they cannot be preferred over conventional bone scans in the diagnosis of bone metastases (Evola et al., 2017). We herein must be highlighted the undeniable role of nuclear medicine in the fields of diagnosis and treatment of the patient. Bone scans by radioisotopes such as TC_99m are helpful in evaluation of benign and metastatic bone lesions. In this case, the bone scan could detect the localization of primary tumors, define the extent of disease and metastasis, and monitor response to treatment. It is worth mentioning that bone metastases are the usual reason for pain in cancer patients. Beam radiotherapy along with the intravenous prescription of radionuclides such as strontium-89, a calcium analog, can be efficient in treating symptomatic bone metastases. Pain ameliorates in 85% of patients and achieves optimal relief (Kramer-Marek and Capala, 2012).

Cancers of the bone and soft tissue, such as osteosarcoma, chondrosarcoma, merkel cell carcinoma, and leiomyosarcoma, have also been implicated with metastasis to the pancreas. However, ES metastasis to the pancreas is considered very rare. Endoscopic ultrasound (EUS) is both effective and safe for diagnosing pancreatic metastasis (Anderson et al., 2020). Furthermore, utilizing of IHC can confirm the suspected diagnosis of pancreatic metastasis (Kandukuri et al., 2017).

Based on the literature searches, 43 cases of ES, including Ewing's sarcoma of bone (ESB), extraskeletal Ewing sarcoma (EES), and PNET involving the pancreas (either as primary or metastasis), had been reported. Among these, only three pelvic cases appeared to be metastatic. These patients were treated with a combination of surgical resection, chemotherapy, and radiation. Disease progression and outcome data were unavailable in one of these three cases, as reported by Saifuddin et al. (2000). The overall prognosis was, however, poor with two of the other reported metastatic cases resulting in death from the disease (Anderson et al., 2020).

In general, approximately 20–30% of ES patients present with metastases at the time of their diagnosis. Multidisciplinary treatment modalities have dramatically improved the 5-year survival rate of patients from 16 to 75% (Fan et al., 2017). Beam irradiation can treat non-resectable initials cancer and chemotherapy can suppress micro metastasis and reduce tumor load before surgery (Anderson et al., 2020).

In our case, the patient did not have metastatic lesions. Consequently, she was treated successfully with trimodality therapy including combined radiation and chemotherapy. The patient until now has remained disease free 4 years after her diagnosis. The patient returned to pain free ambulation without any shortcomings in terms of diagnosis, evaluation, follow up, treatment, and/or quality of life post treatment except for slight limping. Angervall and Enzinger in 1975 announced the first case of extra skeletal ES (Angervall and Enzinger, 1975). Askin in 1979 reported invasive small round cell tumor of the thoraco-pulmonary area and PNET of bone (Askin et al., 1979). Later in 1999, Kollender reported two patients involving periosteal ES (PES) of long bones (Kollender et al., 1999). In 2003 Singh reported a well-developed case of PES (Singh et al., 2003). Kurien in 2015 reported cases with progressive naso-maxillary destruction, proptosis of eye and declined nasal airway capacity (Kurien et al., 2015). It should be noted, in the case series, there are only a few cases of malignancy relating to Ewing sarcoma of the hip and many of them were initially misdiagnosed as septic arthritis (Halwai et al., 2009). de Gauzy et al. announced nine pediatric patients in whom leukemia manifested with a limp due to irritable hip or knee, associated with a raised temperature and anemia (de Gauzy et al., 2014). A recent study on 104 cases with primary Ewing sarcomas of hip reported with three-year event-free survival (EFS) of 32% and overall survival (OS) of 42%. Patients with metastasis only to the bone marrow had an EFS of 52%, whereas those with more than five skeletal metastatic lesions had an EFS of just 16% (Guder et al., 2020). Another study showed that 43% of patients who received ifosfamide and etoposide combination with methotrexate had three-year event-free survival and did not require supplemental doxorubicin or cisplatin (Uezono et al., 2020). However, amendments to interval-compacted five drug chemotherapy are expected, but novel treatment approaches targeting the pathogenic molecular pathways underlying ES will be directed into the clinic in the foreseeable future as well. Increased cooperation among clinical collaborative groups and industry and collaborative groups is necessary to further improve ES prognosis (Gaspar et al., 2015).

## 4 Conclusion

Ewing sarcoma is a very aggressive tumor with variable prognosis relying on stage, site, size, microscopic feature, and biological characteristics. Prognosis has ameliorated thanks to modern chemotherapy and radiopharmaceuticals advancements. The classical, clinical and radiological manifestation of hip area in ES may not be the rule; one should be highly suspicious of the disease even if there is no direct indication to the disease as was encountered in our case. In addition, ES may be misdiagnosed as benign disorders. ES cases of the tibia may mimic as osteomyelitis. ES may manifest as knee monoarthritis, spondylolisthesis, and as postpartum back pain. ES of ilium mimic inflammatory hip arthritis or sacroiliitis. Orthopaedicians, rheumatologists and radiologists should be conscious to this infrequent incidence.

## Abbreviations

EFS: Event-free survival
ES: Ewing's sarcoma
IE: Ifosfamide and etoposide
IHC: Immunohistochemistry
OS: Overall survival
ROM: Range of motion
EUS: Endoscopic ultrasound

## Declaration of competing interest

The authors declare that they have no known competing financial interests or personal relationships that could have appeared to influence the work reported in this paper.
